# Polymyxin B-direct hemoperfusion therapy improves mean arterial pressure in septic shock

**DOI:** 10.1186/cc12940

**Published:** 2013-11-05

**Authors:** Mari Yokota, Ahmin Kim, Taijiro Goto, Tomoyuki Harada, Munekazu Takeda, Mizuho Namiki, Arino Yaguchi

**Affiliations:** 1 Department of Critical Care and Emergency Medicine, Toyo Women's Medical University, Tokyo, Japan

## Background

In our previous study, we reported that polymyxin B-direct hemoperfusion (PMX-DHP) (Toraymyxin^®^; Toray Medical Co., Tokyo, Japan) therapy could contribute to oxygen delivery due to improved hemodynamic status, while decreasing inotropic agents in septic patients immediately after that treatment. The randomized controlled studies are ongoing in other countries, because its efficacy and indication are still controversial issues. The purpose of this study is to evaluate whether PMX-DHP therapy sustains to improve hemodynamic status after the treatment.

## Materials and methods

All adult patients treated with PMX-DHP and receiving a pulmonary arterial catheter (PAC) in our ICU from July 1994 to June 2010 were included in this retrospective observational study. Patients' clinical, microbiological and PAC data were collected from medical archives. PAC variables were compared between immediately before and after 24 hours of PMX-DHP therapy. Values were expressed as mean ± SD. Data were analyzed by Wilcoxon signed-rank test. *P *< 0.05 was considered statistically significant.

## Results

There were 63 patients (36 men, 27 women; age mean 63.4 ± 14.8) studied. The mortality rate was 30.2% 28 days after PMX-DHP. APACHE II score and SOFA score on the day of PMX-DHP therapy were 20.2 ± 14.8 and 7.3 ± 3.8, respectively. Mean arterial pressure (MAP) (mmHg) was significantly increased after PMX-DHP therapy (77.5 ± 22.5 vs. 87.2 ± 15.9, *P *= 0.02). The inotropic score decreased 24 hours after PMX-DHP, but did not reach statistical change (10.0 ± 16.1 vs. 6.3 ± 11.6, *P *= 0.08). The cardiac index (CI) (l/minute/m^2^), systemic venous resistance index (SVRI) (dyn·second·m^2^/cm^5^), mixed venous oxygen saturation (SvO_2_) (%), oxygen delivery and consumption (DO_2 _and VO_2_) (ml/minute) and P/F ratio were not statistically different before and after PMX-DHP therapy.

## Conclusions

Only the increasing of MAP was sustained after 24 hours of PMX-DHP therapy, while the inotropic agents were decreased. Although the CI, DO_2_, VO_2_, and P/F ratio were improved immediately after PMX-DHP therapy in our previous study, these were not significantly changed between before and after 24 hours. PMX-DHP could improve MAP with decreasing inotropic agents, while alterations of other PAC variables were not sustained in 24 hours of PMX-DHP.

